# Conditions for uptake of evidence-based knowledge in municipal care for older people in Sweden: a developmental evaluation

**DOI:** 10.1186/s13104-022-06131-y

**Published:** 2022-07-07

**Authors:** Sara Hultqvist, Lisa Ekstam, Janicke Andersson, Maria H. Nilsson, Marieclaire Overton, Magnus Zingmark, Susanne Iwarsson

**Affiliations:** 1 Department of Health Sciences, Lund University, P.O. Box 157, 221 00 Lund, Sweden; 2 Department of Social Work, Linnaeus University, Universitetsplatsen 1, SE-352 52 Växjö, Sweden; 3 Department of Social Work, Halmstad University, Box 823, 301 18 Halmstad, Sweden; 4 Clinical Memory Research Unit, Department of Clinical Sciences Malmö, Lund University, Lund, Sweden; 5 Memory Clinic, Skåne University Hospital, Malmö, Sweden; 6 Division of Geriatric Medicine, Department of Clinical Sciences, Skåne University Hospital, Lund University, Jan Waldenströms gata 35, building 28, 205 02 Malmö, Sweden; 7 Health and Social Care Administration, Municipality of Östersund, 83182 Östersund, Sweden

**Keywords:** Collaboration, Professional competence, Aging, Older adults, Integrated care, Reg. health care professionals

## Abstract

**Objective:**

The objective of this paper is to describe the initial phase of a long-term collaboration initiative between a municipality and the Faculty of Medicine at a university in Sweden. The overall ambition of the collaboration is to strengthen the quality of care for older people. The concrete goal is to equip academically trained registered health care professionals (HCP) with tools for transferring evidence-based knowledge into practice. As municipal healthcare for older people is mainly carried out by staff lacking academic education, reg. HCP are key actors to bring in and consolidate an evidence-based approach in this setting. Developmental evaluation (DE) has been used to evaluate four separate activities in the initial phase. The activities where sequenced in a cumulative design to provide knowledge for further development of adequate tools.

**Results:**

The cumulative design originally planned did not fit the internal logic of the municipality. Therefore, workflow and pace adjustments were made to proceed towards the joint ambition; the creation of fruitful conditions for the uptake of evidence-based knowledge. Long-term collaboration between academia and organizations outside academia demands a sensitive and flexible research approach, recognizing that collaboration implies mutuality and restricts the sovereignty of academia in designing research.

## Introduction

Though care for older people in Sweden is regulated nationally, 290 local municipalities are responsible for the provision. As municipal autonomy is far-reaching, the landscape is scattered. Ongoing transfer of advanced health care from hospitals (run by the regions) to home and residential care (run by the municipalities) urge for a strengthened capacity of municipal health care. Not least noticed during the COVID-19 pandemic, the demands on municipal health care have increased. Still, most municipal health care staff are assistant nurses, and the proportion of academically trained professionals is low (< 10%). Few registered health care professionals (HCP) working in the municipality setting have education on advanced or research levels, and the care provided is characterized by experience-based work.

Integrated health care, as “an overarching term for a broad and multi-component set of ideas and principles that seek to better co-ordinate care around people’s needs” [1], has been an organizational trend for a long time [2]. Notwithstanding, frequent organizational change is the rule rather than the exception in municipal health care [3, 4]. Therefore, our conviction is that the creation of fruitful conditions for the uptake of evidence-based knowledge must be a context-sensitive endeavor where research and different care organizations collaborate. In line with this, generic knowledge, be it as evidence-based interventions or in other forms, must be tailored to the local context [5, 6]. With this as a backdrop, a long-term collaboration between academia and the care administration in a municipality in southern Sweden was initiated.

Inspired by the Knowledge To Action (KTA) framework [6], we set out to explore incentives, hindrances and needs related to competence development for reg. HCP, and suggest a strategy for knowledge translation and implementation of evidence-based municipal health care for older people. We initiated the development of a structure for partnership between academia and the care administration in the municipality, to enable communication and collaboration.

The objective of this paper is to describe challenges and opportunities during the initial 12-month phase of a long-term collaboration between the Faculty of Medicine at a university and the care administration in a municipality in Sweden.

## Main text

### Methods

Emphasizing learning, developmental evaluation (DE) [7] is well suited to evaluate initiatives in their earliest stages of development [8]. Moreover, DE is an appropriate method for progression in existing complex and/or changing organizations. The evaluator is part of a team whose members collaborate to conceptualize, design, and test new approaches in an ongoing process of continuous improvement, adaptation, and intentional change. The evaluator’s primary function is to elucidate team discussions with evaluative data and logic, and to facilitate data-based decision-making in the developmental process [7]. Key features of DE include a tight integration between evaluators, that is, researchers and implementers, and the use of data for continuous improvement [8]. Accordingly, as researchers and authors of this note, we have worked in tight collaboration with representatives for reg. HCP and for the care administration and had an ongoing discussion concerning the evaluation of the activities. In the following, the activity plan set out in the research plan underpinning the collaboration initiative is briefly presented, followed by the Results section where we contrast the plan with the actual outcome.

To set a sustainable structure for the partnership we formed a steering group, a scientific expert group, an external expert group, and a working group. The four groups were constructed to mirror a spectrum of stakeholders, the municipality and the university but also users and for-profit organizations. In total, about 30 persons were involved. The following activities were scheduled to be carried out as joint actions during the initial 12-month phase:

1. **Data retrieval** on competence levels for reg. HCP in the municipality (N = 277);
2. Nine **dialogue meetings** with reg. HCP, managers, and assistant nurses to identify and discuss conditions for evidence-based practice and their needs of competence development;
3. An **online survey** targeting all reg. HCP in the municipality (N = 277) to identify prerequisites for evidence-based practice and needs of competence development; and
4. Identification of the cornerstones for **strategic planning** for knowledge translation and implementation of evidence-based health care in municipality contexts.

Applying DE, for each of the four activities we developed: (a) a descriptive report of the discrepancy between the planned activity and how it turned out and, (b) lessons learned from the developmental process.

### Results

In the result section we describe activities that did not turn out as planned but still enrichened the collaborative and mutual learning processes. Activities 1–3 were to form a base for the development of practice-oriented strategic planning (activity 4) for knowledge translation. Moreover, as our intention was to prime collaboration and knowledge development, the activities required involvement from both parties.

#### Descriptive data of competence levels

We aimed to collect descriptive data from municipality records on reg. HCP formal qualification levels, including characteristics, such as age, sex, year of professional degree and number of years of work experience. However, knowledge of the staff competence levels was not available as we had assumed. Our request served the process as catalyst, and as the municipal care administration wanted this kind of data to be coordinated in the city’s administrative system, they initiated discussions with the digitization department. Thus, after close to one year after the start of the collaboration a satisfactory solution was not found. Instead, the creation of a simplified register which later could be coordinated in the municipal administrative system was started. Administrators and managers were asked to fill out information on formal qualification levels among reg. HCP.

#### Dialogue meetings

Two rounds of dialogue meetings with different categories of staff (nurses, occupational therapists/physiotherapists, assistant nurses, and managers) and one joint dialogue meeting addressing all four categories were planned. The aim was to promote the reflection on one's own practice and facilitate a dialogue between categories of staff. All reg. HCP and managers received an invitation by email, while the assistant nurses were recruited through the managers. Striving to include twelve representatives from each category of staff, the dialogue meetings aimed at promoting organizational learning. However, fewer persons than expected participated. We ended up with seven dialogue meetings, carried out with four staff categories: one mixed group of occupational therapists and physiotherapists (n = 15), one group of nurses (n = 3), one of managers (n = 5) and one of assistant nurses (n = 7). The first three groups met twice in digital meetings, and the assistant nurses met once in a physical meeting. The recruitment challenges, the varying number of participants and comments from those who did take part indicated that some categories of staff perceived that they had less opportunity to prioritize organizational learning over direct care work. Thus, this experience was brought up with the managers as an obstacle for the uptake of evidence-based knowledge. Due to the low participation rate in the category-specific dialogue meetings, the joint meeting with all participants was postponed to the second year of the collaboration.

#### Online survey

The descriptive data of competence levels (activity 1) and the conversations in the dialogue meetings (activity 2) were to lay the foundation for the development of a survey aimed to identify prerequisites for evidence-based practice and specific need of competence development. We planned for an online survey targeting all reg. HCP in the municipality (N = 277) and thereby diversify and elaborate the empirical materials. However, fewer participants in the dialogue meetings than expected made us rethink using a cumulative research design when building the online survey. Thus, we partly based the survey on findings from the dialogue meetings but also decided to base the survey questions on established questionnaires [9–11] including background questions and questions on attitudes and behavior realted to competence development and research utilization. The questionnaire was piloted and will be administered as a survey online as a first activity in the second year.

#### Cornerstones for strategic planning

To sum up the findings and feed into a process to identify the cornerstones for strategic planning, a meeting involving all the four groups was scheduled as an activity towards the end of the first phase of the collaboration. The rationale was to initiate strategic planning, to be used as a tool for the development of concrete measures supporting the uptake of evidence-based knowledge in municipal care for older people. However, as such a process required empirical and experiential input from previous activities [1–3] and as only eleven of 22 invited participants signed up for the workshop, the aim of the meeting was reconsidered. Thus, the meeting did not produce any concrete basis for strategic planning but created an arena for collective reflection. The empirical results from the dialogue meetings and the weak representation were discussed. The care administration's visions of participating in research and pursuing issues of increased competence and evidence-based practice were brought up and shared in the light of the joint experiences made this far. This discussion of visions, missions, goals and challenges will influence the continuation of the collaboration.

### Discussion

In this initial phase of a long-term collaboration, we used DE to highlight and learn from planned activities that did not turn out as expected. As one research task in DE is to facilitate data-based decision-making developmental processes [7], we have surely contributed by highlighting the lack of a register of staff characteristics and competence. Moreover, by making the managers aware of the poor attendance at the dialogue meetings, we have elucidated the fact that collaboration tends to remain on a rhetoric level if the intended group (i.e., HCP) does not participate. New insights into the municipal organization have made us aware of the conflicting requirements the HCP must handle.

If the visions and ambitions agreed on by managers and scholars are to imbue care practice, anchoring is required. For example, anchoring the decision to set-up the register to achieve an overview of the competence level among the staff and, anchoring the view that engagement in research is a work task among others. The high frequency of organizational change initiatives in the municipal health care sector [3] probably causes ‘project fatigue’ and feelings of disengagement among staff. This might be one explanation for why things turned out differently than planned.

The postponement of the joint dialogue meeting, the revised methodology to develop the online survey and the redefinition of the strategic planning process could be interpreted as failures in relation to the original plan. Interpreted as a lesson for the future, these outcomes advocate a flexible research approach, recognizing that collaboration implies mutuality and restricts the sovereignty of academia in designing research, a *reciprocal logic.*

Applying DE, the authors of this note are researchers and actors in the process as well as evaluators, and the implementers are reg. HCP and managers. The KTA framework pinpoints the fact that when we as scholars speak of knowledge, we primarily refer to scientific research [6]. The KTA process occurs in a complex social system of interactions among stakeholders, but the knowledge per se is epistemologically derived from scientific analysis. As a matter of fact, the question “What do we mean when we talk of knowledge and evidence?” spontaneously came up during meetings between researchers and municipality staff. This discussion reflected the importance of clarifying perspectives on evidence-based practice.

To conclude, if collaboration is to be a truly mutual enterprise, academia must be prepared to confer power to the partnering organization. This transfer of power reduces the academic control of the course of events. Planned activities can easily be overturned by factors belonging to the partnering organization [12–14]. Such ‘extended uncertainty’ is certainly more demanding than a working mode where only researchers are in charge.

## Limitations

As the authors of this note all are researchers, no one representing the municipality has co-authored this piece. Even if none of the persons involved in the groups fulfilled the criteria required for scientific authorship, the content was strongly influenced by their input during meetings. For transparency, the content of this paper was shared with several representatives from the municipality prior to submission.

## Data Availability

Data collected for this research note will, after de-identification, be available on reasonable request after publication in peer reviewed journals. The prerequisite for this is a data transfer agreement, approved by legal departments of the institutions of both the requesting researcher and the researchers that provided data for the study. Proposals should be directed to: sara.hultqvist@med.lu.se.

## Abbreviations

DE: Developmental evaluation
HCP: Health care professionals
KTA: Knowledge to action
